# Bioenergetics of fish spermatozoa with focus on some herring (*Clupea harengus*) enzymes

**DOI:** 10.1007/s10695-019-00650-5

**Published:** 2019-05-20

**Authors:** J. Gronczewska, N. Niedźwiecka, K. Grzyb, E. F. Skorkowski

**Affiliations:** Department of Molecular Evolution, Faculty of Biology, University of Gdańsk, 80-308 Gdańsk, Poland

**Keywords:** Herring spermatozoa, Metabolic regulation, ME, CK, LDH

## Abstract

Herring (*Clupea harengus*) shows the unique behavior of reproductive biology in which spermatozoa remains in the surrounding media for extended periods. It is an excellent model for studying the malic enzyme (ME) and creatine kinase (CK) biochemical properties because of their high activity and variability of molecular isoforms. The specific activity of NAD-preferring ME in herring spermatozoa is the highest among other fish spermatozoa and is localized in its large mitochondrion. Two different CK isoforms, dimer and octamer, were detected in herring spermatozoa. It has already been shown that CK isoforms play an important role in energy homeostasis by catalyzing a reversible transfer of the phosphate of ATP to creatine to yield ADP and creatine phosphate (CP) (creatine/CP circuit). Two lactate dehydrogenase (LDH) isoenzymes were also shown in herring spermatozoa, LDH-B_4_ and LDH-A_2_B_2_. In this mini-review, the role of ME and energy transport system with easily diffusible creatine and CP in herring spermatozoa is discussed.

## Introduction

Earlier studies suggest that the characteristics of progressive forward motility of spermatozoa were related to their fertilizing capacity and the sperm motility which was dependent on ATP content (Christen et al. 1987; Perchec et al. 1995). ATP provides energy for spermatozoa movement, and this energy is utilized by the dynein ATP-ase that is localized within the flagellar motile apparatus, the axoneme (Inaba et al. 1999; Inaba 2008). There is no doubt that maintenance of high level of ATP in spermatozoa is important for modulating flagellar motility. It is well established that ATP depletion causes decrease of rainbow trout (*Salmo gairdneri*) and carp (*Cyprinus carpio*) spermatozoa movement (Christen et al. 1987; Perchec et al. 1995) and that creatine phosphate (CP) can stimulate sperm motility in the rainbow trout spermatozoa (Saudrais et al. 1998). ATP concentration in semen of Chinook salmon (*Oncorhynchus tshawytscha*) and steelhead trout (*Oncorhynchus mykiss*), kept in the absence of oxygen, decreased to approximately 10% of initial values within 8 h and remains unchanged through the following 64 h (Bencic et al. 1999a, b). Taken together, these results suggest that experimental conditions can influence ATP concentration and motility of fish spermatozoa. Fish spermatozoa contain glycolytic pathway, Krebs cycle (tricarboxylic acid cycle) and oxidative phosphorylation as key pathways contributing to ATP production (Mansour et al. 2003). Recently, all metabolic processes as glycolysis, mitochondrial respiration, and oxidative phosphorylation were confirm in carp spermatozoa by electrophoresis and liquid chromatography (not native methods) connected with mass spectroscopy (Dietrich et al. 2014, 2016) and rainbow trout (Nynca et al. 2014). More details about metabolism of fish spermatozoa was reviewed earlier (Lahnsteiner 2008).

Very little is known about the enzymes and their biophysical and kinetic properties in fish spermatozoa as compared to activity and expression in somatic tissues. High creatine kinase (CK) activity was observed in herring spermatozoa, where two isoforms displayed different electrophoretic mobility than the isoforms present in its skeletal muscle (Grzyb et al. 2003). The two different CK isoforms were detected to be a characteristic feature of herring spermatozoa, octamer, and dimer and these isoforms are not expressed in other herring tissues (Grzyb and Skorkowski 2006). Two lactate dehydrogenase (LDH) isoenzymes were shown in herring spermatozoa, LDH-B_4_ and LDH-A_2_B_2_ (Gronczewska et al. 2003). The spermatozoa from herring show the presence of two molecular forms of malic enzyme (ME), higher activity of the NAD-preferring ME and low activity of the NADP-specific ME (Niedźwiecka and Skorkowski 2013). Specific activities of enzymes involved in the total generation of NADPH [isocitrate dehydrogenase (IDH), ME and glucose-6-phosphate dehydrogenase (G-6-PDH)] are higher in herring spermatozoa than in carp (*Cyprinus carpio*) and catfish (*Clarias gariepinus*) spermatozoa (Gronczewska et al. 2003).

Mitochondria of eukaryotic cells play a fundamental role in metabolism and ATP synthesis through oxidative phosphorylation. The fine structure of the fish spermatozoa showed that Clupeid possess the head surrounded by one large mitochondrion and demonstrates that there is a considerable similarity in spermatozoan ultrastructure in these fish (Gwo et al. 2006; Ulloa-Rodriguez et al. 2017). The consistency in the general morphology of clupeid sperm indicates that the order is a cohesive unit (Gwo et al. 2006; Ulloa-Rodriguez et al. 2017). Piomboni et al. (2012) did not show participation of ME in review about the role of mitochondria in bioenergetics of mammalian spermatozoa. They also did not show participation of the energy transport system with easily diffusible creatine/creatine phosphate circuit that was described earlier by Tombes and Shapiro (1987) for sea urchin spermatozoa and Schlegel et al. (1990) for chicken heart tissues. This mini-review will discuss the role of some enzymes involved in bioenergetics of herring spermatozoa.

## Malic enzyme (ME)

ME catalyzes the reversible decarboxylation of malate to form pyruvate in the presence of coenzyme NADP or NAD and the divalent cation Mn^2+^ or Mg^2+^:

$$ \mathrm{Malate}+\mathrm{NAD}\left(\mathrm{P}\right)\overset{{\mathrm{Mn}}^{2+}}{\leftrightarrow}\mathrm{Pyruvate}+{\mathrm{CO}}_2+\mathrm{NAD}\left(\mathrm{P}\right)\mathrm{H} $$

Various molecular forms of ME are classified based on relative affinity for coenzymes and their ability to decarboxylate oxaloacetate. The full name according to nomenclature and classification of enzyme is: NAD(P) oxidoreductase (decarboxylating), (EC 1.1.1.38, 1.1.1.39, 1.1.1.40). Depending on the species and type of tissue ME may occur in the cytosol, mitochondria or both cellular compartments simultaneously (Frenkel 1975). Enzymes isolated from the two subcellular compartments have different catalytic and biophysical properties.

With very few exceptions, the ME of mammals is NADP-dependent. The first to show that mitochondria isolated from calf adrenal gland and rabbit heart have two different molecular forms of ME, NADP-dependent and other that use NAD and NADP as coenzyme (Sauer 1973; Lin and Davis 1974). The mitochondria of cod (*Gadus morhua*) and brown trout (*Salmon trutta*) hearts along with herring skeletal muscle, liver, and testicular tissues have two forms of ME (Skorkowski et al. 1984, 1985; Biegniewska et al. 1990) with properties like the enzymes present in the mitochondria of rabbit heart (Lin and Davis 1974). The lower molecular weight form is selectively specific for NADP as the coenzyme, whereas the higher molecular weight form uses both coenzymes but shows a higher activity in the presence of NAD (thus called NAD-preferring). The native forms of the two mitochondrial enzymes can also be separated by electrophoresis on polyacrylamide gels (Skorkowski et al. 1985). Both mitochondrial forms migrate towards the anode but the NADP-specific form is the slower migrating enzyme.

**Fig. 1:**
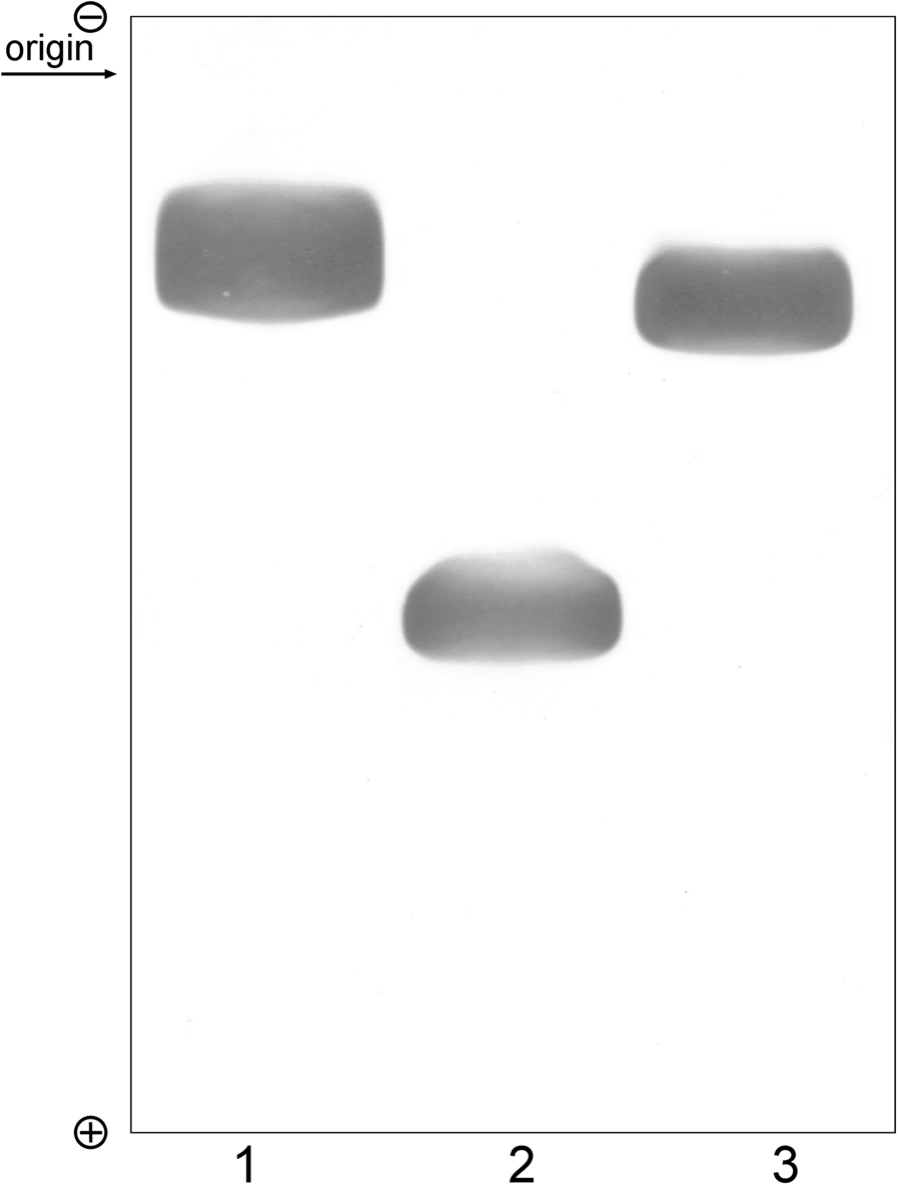
Polyacrylamide gel electrophoresis of three forms of malic enzyme from herring (*Clupea harengus*) skeletal muscle: cytosol NADP-specific ME (1); mitochondrial NAD-preferring ME (2), and mitochondrial NADP-specific ME (3). Sample of purified enzymes were applied in 10 μl aliquots to each gel. Electrophoresis was run for 10 h at 2 °C. All gels were stained for malic enzyme activity as described (Skorkowski et al. 1985)

Separation by PAGE of the three MEs from the herring skeletal muscle under native conditions is shown in Fig. 1. The two mitochondrial MEs migrated as distinct single bands towards the anode, slower migrating enzyme being the NADP-specific form. These results agree with the fact that the higher ionic strength was required for the elution of NAD-preferring enzyme from the DEAE-Sephacel then for the elution of NADP-specific enzyme (Biegniewska et al. 1990). The cytosol NADP-specific ME is the slowest migrating enzyme.

Molecular forms of ME present in the cell had different biophysical properties. Some of the biophysical properties and reversibility of the reaction catalyzed by three different forms of ME in the heart of the salmon trout (*Salmo trutta*) are listed in Table 1 (Skorkowski 1988). Of note is the ability of ME to catalyze the pyruvate carboxylation reaction. The NADP-specific mitochondrial ME (Biegniewska and Skorkowski 1987) readily catalyzes this reaction but this property is lacking in the NAD-preferring mitochondrial enzyme. The results presented here suggest fish mitochondria having two forms of ME, one NADP-specific and other NAD-preferring. NADP-specific ME in mitochondria has comparable properties to cytosol NADP-specific ME, e.g., electrophoretic mobility (Fig. 1), catalytic properties and reductive carboxylation of pyruvate to malate (Table 1). The possible role of pyruvate carboxylation by the mitochondrial ME in the fish heart may aid in the synthesis of the Krebs cycle intermediates during the oxidation of fatty acids (Bilinski 1974).

**Table 1:** Physico-chemical properties and reversibility of the reaction catalyzed by three forms of malic enzyme from salmon trout (*Salmo trutta*) heart (Skorkowski 1988)

Enzyme	Molecular weight	Isoelectric point	pH	% of forward reaction
Mitochondrial NADP-specific EC 1.1.1.40	190,000	5.85	6.5 7.0 7.5	376 100 13
Mitochondrial NAD preferring EC 1.1.1.39	280,000	5.45	6.5 7.0 7.5	0 0 0
Cytosol NADP-specific EC 1.1.1.40	290,000	5.1	6.5 7.0 7.5	800 162 35

In herring tissues, various molecular forms of ME may occur, both in the cytosol and mitochondria (Fig. 1). Herring spermatozoa exhibit a higher specific activity of ME than salmon, trout, carp, and catfish spermatozoa (Niedźwiecka and Skorkowski 2013). The spermatozoa from herring show the presence of two molecular forms of ME. Both ME forms were separated from herring spermatozoa by chromatography on DEAE-Sepharose. Herring spermatozoa contained higher activity of the NAD-preferring ME and low activity of the NADP-specific ME (ratio about 33:1) (Niedźwiecka and Skorkowski 2013). High activity of ME suggests adaptation of herring spermatozoa to metabolism at high oxygen tension for herring spawn.

Table 2 presents the specific activity of ME in mitochondria isolated from various species. It is worth noting that the activity of ME in aquatic animals is much higher than in terrestrial animals. The distribution of ME activity in mitochondria isolated from different herring tissues and spermatozoa in the presence of the coenzyme NADP and NAD are shown in Table 2. The highest specific activity per milligram mitochondrial protein is found in the testes of herring—256 nmol/NADPH/min per mg and is comparable to the muscle of the American crayfish. The activity of NAD-preferring ME in herring spermatozoa is about 356 nmol NADH/min per mg (Niedźwiecka and Skorkowski 2013). The activity of ME in mitochondria isolated from ovaries was the lowest of herring tissues. Mitochondria isolated from herring testes had high specific activity of ME. The specific activity of ME per mg of testis mitochondrial protein was more than 30 times higher than that of ME in ovarian mitochondria and more than three times that of ME activity in liver and skeletal muscle mitochondria (Table 2).

**Table 2:** Identification of mitochondrial malic enzyme activities from various species

Specific activity (nmol/min per mg mitochondrial protein)			
Source of mitochondria	NADP-dependent	NAD-dependent	References
Crayfish (abdomen muscle)	230	N.D.	Skorkowski et al. (1977)
Cod (heart)	160	26	Skorkowski et al. (1984)
Salmon trout (heart)	90	54	Skorkowski et al. (1985)
Herring (skeletal muscle)	74.8	37	Biegniewska et al. (1990)
(ovaries)	8.4	N.D.	Biegniewska et al. (1990)
(liver)	70.7	62	Biegniewska et al. (1990)
(testes)	256.3	387	Biegniewska et al. (1990)
(spermatozoa with large mitochondrion)	213	356	Niedźwiecka and Skorkowski (2013)
Rabbit (heart)	13	49	Skorkowski et al. (1984)
Rat (heart)	40	N.D.	Skorkowski et al. (1984)
(skeletal muscle)	30	N.D.	Świerczyński (1981)
(liver)	0.5	N.D.	Moreadith and Lehninger (1984)
(hepatoma)	32	N.D.	Moreadith and Lehninger (1984)
Mouse (hepatoma)	82	N.D.	Moreadith and Lehninger (1984)

**Fig. 2:**
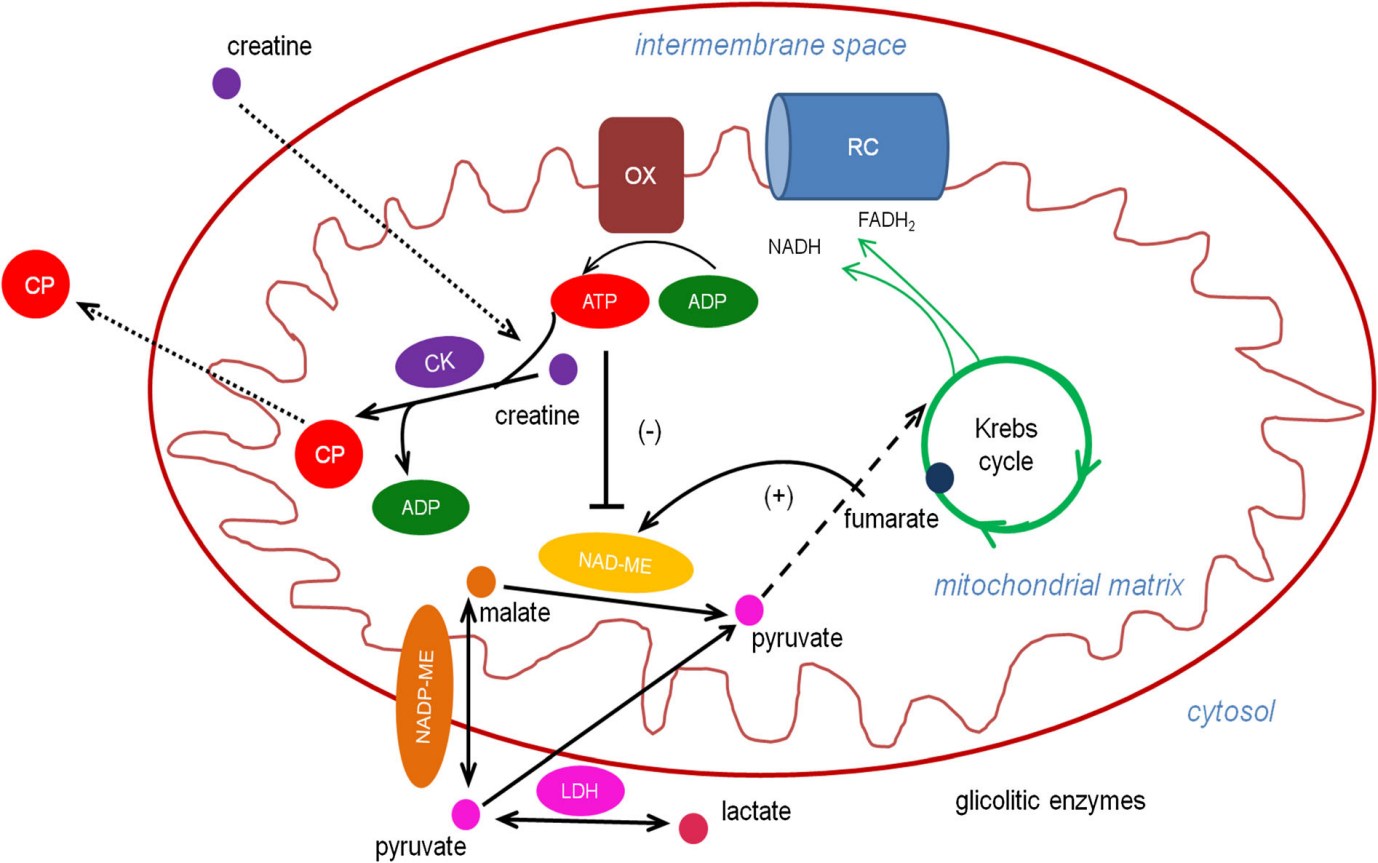
A schematic mitochondrion and role of malic enzyme (ME) and creatine kinase (CK) in energy buffering in herring spermatozoa. Mitochondrial octamer and cytosolic dimer isoforms of CK are associating with ATP-providing and ATP-consuming (e.g., the dynein ATP-ase) processes. NADP-ME carboxylate pyruvate to malate. NAD-ME decarboxylate malate to pyruvate and generate NADH. NAD-ME is competitively inhibited by ATP. Fumarate reversed ATP-dependent inhibition of NAD-ME. ATP level and in consequence ME activity is under control of creatine kinase. OX, oxidative phosphorylation; CP, creatine phosphate; RC, respiratory chain

Localization of ME in mitochondria is important because these organelles are involved in energy production by oxidative phosphorylation (Fig. 2). NAD-preferring ME from herring spermatozoa was localized in the mitochondrion by an antibody raised in rabbit against NAD-preferring ME isolated from herring spermatozoa (Niedźwiecka et al. 2017). ATP was found to decrease the rate of NAD reduction of NAD-preferring ME from herring spermatozoa with respect to malate as was shown earlier. Two-millimolar ATP reduces the rate of NADH formation to about 10% compared to control conditions (NAD-preferring ME without inhibitor addition) in the presence of 5 mM malate (Niedźwiecka et al. 2017). On the other hand, fumarate was very effective in reversing ATP inhibition of NAD-preferring ME from herring testicular tissue and in the presence of 2 mM fumarate, ATP inhibition was reversed to about 70% of the activity in the absence of ATP (Skorkowski and Storey 1990). Inhibition of NAD-preferring ME could be overcome by fumarate and in consequence could adjust spermatozoa metabolism to changing levels of ATP and fumarate (Niedźwiecka et al. 2017). Previously, it has been shown that ATP is an effective inhibitor of both NAD- and NADP-linked reaction by NAD(P)-ME from mammals and fish (Sauer 1973; Lin and Davis 1974; Skorkowski and Storey 1988, 1990; Żołnierowicz et al. 1988).

## Creatine kinase (CK)

Creatine kinase (EC 2.7.3.2) isoforms play important role in energy homeostasis, by catalyzing the reversible transfer of the phosphate of ATP to creatine to yield ADP and creatine phosphate (CP) (Jacobus and Lehninger 1973). In the sea urchin spermatozoa and in most mammalian tissues, cytosolic dimeric CK is coexpressed with an octameric mitochondrial isoform and together with high intracellular concentrations of easily diffusible creatine and CP; these isoenzymes maintain a cellular energy buffer and energy transport system (creatine/CP-circuit) (Tombes and Shapiro 1987; Schlegel et al. 1990). The rainbow trout (*Salmo gairdneri*) spermatozoa showed a very high cytosolic CK activity which was purified and characterized as a main testicular protein (Saudrais et al. 1996). Earlier results showed only one cytosolic CK isoform for teleostean fish spermatozoa (Tombes and Shapiro 1989; Saudrais et al. 1996).

High CK activity was also observed in herring spermatozoa, where two isoforms displayed different electrophoretic mobility than the isoforms present in its skeletal muscle (Grzyb et al. 2003). The two different CK isoforms were detected to be a characteristic feature of herring spermatozoa and these isoforms are not expressed in other herring tissues (Grzyb and Skorkowski 2005, 2006). Isolation and purification procedures allowed obtaining purified enzymes with specific activity of the 345 μmol/min/mg protein for octameric CK and 511 μmol/min/mg protein for dimeric CK. Native molecular weight of the octamer and dimer determined by gel permeation chromatography was about 330,000 and 90,000, respectively (Grzyb and Skorkowski 2006).

Most mitochondrial CK isoforms investigated so far (bovine, chicken, pig, pigeon, rabbit, and rat) form octameric molecules that are composed of eight identical subunits with a molecular weight of about 43 kDa each (Wyss et al. 1992). The molecular weight of CK1 (homooctamer) and CK2 (homodimer) subunits from herring spermatozoa were: about 42 and 43 kDa, respectively (Grzyb and Skorkowski 2006). Similar molecular weight of dimer and octamer subunits is present in spermatozoa of polychaete (Ellington et al. 1998) and herring skeletal muscle (Grzyb and Skorkowski 2005).

Fast dissociation of mammalian octamers within hours and minutes is induced by TSAC (Transition State Analogue Complex), an artificial dimerization complex (Schlegel et al. 1988). The oligomeric transitions of herring spermatozoa octamers could be determined by gel filtration chromatography. The herring spermatozoa octamers incubated with TSAC also caused octamer destabilization and almost all of octamers dissociated into dimers within 8 h at 4 °C (Grzyb and Skorkowski 2006).

Herring tissues represent very high and variable expression of CK isoforms. Most of them are negatively charged and migrate towards anode during native electrophoresis. These CKs isoforms have putative cytosolic origin and form dimeric structures. In herring skeletal muscle, cytosolic CK band migrated towards anode and mitochondrial one towards cathode (Grzyb and Skorkowski 2005). The cytosolic CK from herring skeletal muscle looks very similar in native electrophoretic mobility to higher vertebrates muscle-type CKs (Ishida et al. 1991) and to CK-A from green sunfish (Fisher and Whitt 1978). The muscle-type CK has a wide distribution in herring tissues and was also found in the herring eye, stomach, gill, and heart (Grzyb and Skorkowski 2006). In the herring brain and eye extract, we detected the most anodal isoform from all cytosolic CKs, it is probably brain-type CK and the other isoform which is suggested to be heterodimer consisted with muscle-type and brain-type monomers (Grzyb and Skorkowski 2006). We also noted a significant activity of positively charged CK isoforms. These isoforms were found in spermatozoa, eye and stomach as well as in skeletal muscle. These positively charged CK isoforms are mitochondrial octamers.

The CK activity is the highest in spermatozoa (Grzyb et al. 2003) and the two different CK isoforms, octamer and dimer, were detected that are characteristic for sperm only and not expressed in other herring tissues. It was shown earlier that cytosolic CK from trout spermatozoa is different from that ones in somatic tissues (Saudrais et al. 1996). These findings make these isoforms an interesting model for studies of the fish CK biochemical properties.

It is known that CK isoforms differ in amino acid sequence and immunological properties (Roberts and Grace 1980). Using our polyclonal anti-CK2 antisera (herring sperm homodimer CK), a positive response was obtained only with pure CK2 protein (Grzyb and Skorkowski 2006). No response was detected with octameric CK protein neither from herring spermatozoa nor with pure cytosolic dimer and mitochondrial octamer from herring skeletal muscle (Grzyb and Skorkowski 2006). These results correspond with specific staining for CK activity (Grzyb et al. 2003; Grzyb and Skorkowski 2006) and support the assumption that octameric CK1 and dimeric CK2 are spermatozoa-specific proteins.

## Lactate dehydrogenase (LDH)

In fish spermatozoa during motility and during immotile storage, glucose levels decreased. When respiratory activity is inhibited, the levels of lactate increase (Lahnsteiner et al. 1992, 1993). Pyruvate is formed during glycolysis and from catabolism of some amino acids. It has been shown that pyruvate could stimulate spermatozoa motility and viability when added as substrate to the incubation medium (Lahnsteiner et al. 1999). The authors also noticed that respiration and glycolytic rates increase significantly during motility. In fish and other vertebrates, lactate dehydrogenase (EC 1.1.1.27) is a tetramer consisting of subunits coded by three independent loci. This enzyme catalyzes the interconversion of pyruvate and lactate. LDH isoenzymes differ from each other and exhibit distinct tissue expression (Markert et al. 1975; Ziętara and Skorkowski 1993). It was shown earlier that there are only two LDH isoenzymes in herring spermatozoa, LDH-B_4_ and LDH-A_2_B_2_. These two isoenzymes are also present in herring heart. These isoenzymes possess different biophysical properties, pH optima for substrates, different thermostability (Gronczewska et al. 2003). The presence of the LDH-B_4_ and LDH-A_2_B_2_ isoforms normally suited better for aerobic conditions conforms well to the Lahnsteiner et al. (1992, 1993, 1999) findings. The activity of LDH in herring spermatozoa was very low. On the other hand, LDH activities in spermatozoa of carp (*Cyprinus carpio*) and catfish (*Clarias gariepinus*) living in less anaerobic water were about 14 and 10 times higher than in herring spermatozoa (Gronczewska et al. 2003). It was shown also that spermatozoa from African catfish (*Clarias gariepinus*) during long-term storage at 4 °C in the presence of lactate is the most favorable substrate to maintain ATP concentration and physiological level of adenylate energy charge (Ziętara et al. 2004; Gronczewska and Skorkowski 2019 accepted for publication).

## Role of ATP in fish spermatozoa motility

The duration of fish spermatozoa motility in the natural environment varies greatly among fish species from seconds to days. This large difference probably depends on the capacity of the spermatozoa to restore intracellular ATP and creatine phosphate (CP) concentrations. Fish spermatozoa during motility have very high energy turnover and consuming large amounts of ATP to maintain gradients between mitochondria and the flagella.

The adenylate energy charge (AEC = [ATP] + 1/2 [ADP]/[ATP] + [ADP] + [AMP]) has been proposed as the marker of energy store in the adenine nucleotide pool of living cells (Atkinson 1968). Many papers have confirmed that the physiological AEC of somatic cells and spermatozoa ranges from 0.8 to 0.95 (Atkinson 1977; Dreanno et al. 1999; Smoleński et al. 1990; Ziętara et al. 2004). It has been shown that after activation of rainbow trout and turbot spermatozoa, the intracellular ATP concentration decreased rapidly and ADP and AMP concentrations increased (Christen et al. 1987; Dreanno et al. 1999). Simultaneously, the AEC value declined from 0.95 before motility initiation and continued to decrease during spermatozoa swimming and after 60 s the AEC value is about 0.5. Already 10 min after spermatozoa activation, CP disappeared in turbot spermatozoa (Dreanno et al. 1999). It has been shown that African catfish spermatozoa possess a rather low AEC (approximately 0.55) and display a relatively high rate of adenine catabolism which leads to ATP depletion (Mansour et al. 2003; Rurangwa et al. 2002; Ziętara et al. 2004). In contrast, AEC value of carp spermatozoa kept at identical conditions was 0.96 indicating that carp spermatozoa showed a remarkable capability for stabilizing the AEC value (Rurangwa et al. 2002; Biegniewska et al. 2010).

ATP formed in mitochondria by oxidative phosphorylation can transfer its terminal high-energy phosphoryl group to form chemical compounds of relatively similar high-energy character like CP. This process is catalyzed by mitochondrial CK octamer according to the reaction: ATP + creatine → ADP + CP (Fig. 2). When intracellular concentration of ATP decreases, cytosol CK dimer catalyzes the reaction: ADP + CP → ATP + creatine, to prevent further decrease of ATP concentration and/or to restore ATP level close to physiological value. In this way, CK plays a key role in energy metabolism of many cells. It is well established that CP can support rainbow trout sperm motility (Saudrais et al. 1998). Recently, in review about cellular compartmentation of local energy circuit of ATP generated by oxidative phosphorylation and consumption, more attention were directed to role of CK isoforms (Schlattner et al. 2016). In mitochondria, ATP generated by oxidative phosphorylation is associated with CK octamer enabling the direct transfer of energy from ATP to creatine and form CP. The major part of ATP utilization is linked to dynein, precisely the dynein ATP-ase localized within the flagellar motile apparatus, the axoneme (Inaba et al. 1999; Inaba 2008). This suggests that CK also plays an essential role in fish spermatozoa energetic metabolism. The mitochondrial CK isoform is functionally coupled with an ATP-providing process in oxidative phosphorylation and with easily diffusing CP through the voltage-dependent anion channel at outer mitochondrial membrane could support cytosol ATP-consuming processes (for review, see Schlattner et al. 2016). Interestingly, the ATP concentration is several folds lower than that of CP in fish spermatozoa (Rurangwa et al. 2001). CP is an alternative energy carrier and compared to ATP is much smaller in molecular size and is significantly more diffusible than ATP.

## Discussion

Upon spawning, Pacific herring (*Clupea pallasii*) spermatozoa show the unique behavior of reproductive biology in which male fish spawn first, with spermatozoa remaining in the surrounding media for extended periods, followed by the spawning of females (Vines et al. 2002). The optimum salinity for fertilization in *C. pallasii* from San Francisco Bay peaked at 16 ppt salinity and from Aristo Sound, Turku, Baltic Sea of *C. harengus* was about at 8 ppt salinity (Griffin et al. 1998). Pacific herring spermatozoa motility is initiated in the presence of sperm motility initiation factor (SMIF), a 105-kDa glycoprotein that is localized to the micropylar region of the herring egg (Griffin et al. 1996). Motility of Pacific herring spermatozoa also could have been initiated by herring sperm-activating peptide (HSAP, molecular mass about 8 kDa) that is released from the eggs at spawning (Oda et al. 1995, 1998). It is likely that this mechanism has evolved in herring *C. pallasii* spermatozoa for regulation of motility initiation until contact with the ligand released from eggs (Vines et al. 2002; Inaba 2008).

Especially high CK-specific activity was observed in herring spermatozoa, where two isoforms displayed a different electrophoretic mobility from the cytosolic homodimer and mitochondrial homooctamer present in its skeletal muscle (Grzyb et al. 2003; Grzyb and Skorkowski 2005, 2006). High activity of CK, and its subcellular localization and regulation by polymerization and depolymerization suggest that CK plays a key role in energetic metabolism of herring spermatozoa (Grzyb and Skorkowski 2006). Tombes and Shapiro (1987) showed that energy produced in mitochondria is required for motility of sea urchin (*Strongylocentrotus purpuratus*) sperm where mitochondrial CK isoenzyme was found. In the sea urchin spermatozoa and in most mammalian tissues, cytosolic dimeric is coexpressed with an octameric mitochondrial isoform of CK. Together with high intracellular concentrations of easily diffusible creatine and CP, these isoenzymes maintain a cellular energy buffer and energy transport system (creatine/CP-circuit) (Tombes and Shapiro 1987; Schlegel et al. 1990). Rainbow trout (*Salmo gairdneri*) spermatozoa showed a very high cytosolic CK activity, which was purified and characterized as a main testicular protein (Saudrais et al. 1996). Earlier results showed only one cytosolic CK for teleostean fish spermatozoa (Tombes and Shapiro 1989; Saudrais et al. 1996). The role of phosphagens in metabolic regulation in cells displaying high and variable rates of aerobic energy synthesis at evolutionary context was reviewed earlier (Ellington 2001).

Localization of ME in mitochondria is important because these organelles are involved in energy production. In herring spermatozoa, mitochondrial NAD-preferring ME was inhibited by ATP and fumarate reversed this inhibition (Niedźwiecka et al. 2017). The physiological concentration of ATP (2–3 mM) in the matrix of respiring mitochondria is sufficient to completely inhibit NAD-preferring ME when ATP is reasonably high, the inhibitory effect of ATP must be overcome. Regulation of NAD-preferring ME activity in vivo might depend on an increase in the concentration of mitochondrial fumarate and also could be activated by creatine or ADP by the CP/creatine ratio and in consequence stimulate respiration in mitochondria. Mitochondrial CK present in herring spermatozoa (Grzyb and Skorkowski 2006) could perform a key role on ATP recycling mechanism and could control of NAD-preferring ME activity (Fig. 2). During the depletion of ATP when fish spermatozoa stop swimming (Christen et al. 1987), inhibition of ME could be also overcome and in consequence metabolism in spermatozoa is enhanced to restore ATP level.

In herring spermatozoa, ME activity is the highest among the NADP-dependent enzymes present in this cell. Main form of ME from herring spermatozoa uses both coenzymes, NAD and NADP, but preferring NAD as coenzyme (Niedźwiecka and Skorkowski 2013). It was suggested that fish mitochondria utilize malate as respiratory fuels and ME may function in the provision of intramitochondrial pyruvate (Skorkowski et al. 1984). ME is particularly interesting since it uses pyruvate as a substrate and provides an alternative route for pyruvate metabolism in fish muscle during active mobilization of protein as an energy source or support gluconeogenesis in the liver. ME showed stability in activity during spawning migration of sockeye salmon (*Oncorhynchus nerka*) (Mommsen et al. 1980; Mommsen 2004).

Two LDH isoenzymes present in herring spermatozoa—LDH-B_4_ and LDH-A_2_B_2_ catalyzes interconversion of pyruvate and lactate (Gronczewska et al. 2003). The presence of the LDH-B_4_ and LDH-A_2_B_2_ isoforms suite better for aerobic conditions agrees well with the adaptation of herring spermatozoa to unusual behavior during spawning.

## Conclusion

Only limited information is available about the properties of purified native enzymes from fish spermatozoa involved in energetic metabolism. Herring (*Clupea harengus*) is an excellent model for studying the fish ME and CK biophysical properties because of high activity and variability of molecular isoforms of enzymes existing in its tissues. Three different molecular forms of ME occur in herring skeletal muscle, one cytosol specific for NADP only as the coenzyme and two in mitochondrion one specific for NADP only and the other utilizing both coenzymes but preferring NAD. High NAD-preferring ME activities were identified in mitochondrial fractions isolated from herring skeletal muscle, liver, testes, and spermatozoa. NAD-preferring ME has allosteric properties. This creates numerous possibilities for metabolic regulation for this form of the enzyme which as opposed to the cytosolic form is also controlled by ATP. The regulation of NAD-preferring ME activity in vivo might depend on the increase in the concentration of mitochondrial fumarate and also could be activated by creatine or ADP by the CP/creatine ratio and in consequence stimulate respiration in mitochondria (Fig. 2). Herring spermatozoa possess two forms of creatine kinases different that in somatic tissues, cytosol dimer, and mitochondrial octamer which are responsible for creatine/CP circuit. CK isoforms and diffusible CP are cellular energy buffers between ATP generation and consumption within cell. Lactate dehydrogenase and malic enzyme both utilized pyruvate as a substrate. The findings discussed in this mini-review give a better understanding of molecular adaptation of herring spermatozoa to unusual behavior during spawning.
